# Langerian mindfulness, quality of life and psychological symptoms in a sample of Italian students

**DOI:** 10.1186/s12955-018-0856-4

**Published:** 2018-02-06

**Authors:** Francesco Pagnini, Katherine E. Bercovitz, Deborah Phillips

**Affiliations:** 1 Department of Psychology, Università Cattolica del Sacro Cuore, Via Nirone 15, 20123 Milan, Italy; 2 Department of Psychology, Harvard University, 33 Kirkland St, Cambridge, MA 02138 USA

**Keywords:** Langerian mindfulness, Langer mindfulness scale, Quality of life, Depression, Anxiety

## Abstract

**Background:**

Noticing new things, accepting the continuously changing nature of circumstances, and flexibly shifting perspectives in concert with changing contexts constitute the essential features of Langerian mindfulness. This contrasts with a “mindless” approach in which one remains fixed in a singular mindset and is closed off to new possibilities. Despite potentially important clinical applications for this construct, few studies have explored them. The instrument developed to measure Langerian mindfulness is the Langer Mindfulness Scale (LMS), although this tool has been limited primarily to English-speaking populations. The study aimed to test LMS validity in the Italian language and to analyze the relationships between Langerian mindfulness and well-being.

**Methods:**

We translated the LMS into Italian, analyzed its factor structure, and investigated the correlation between mindfulness and quality of life and psychological well-being in a sample of 248 Italian students (88.7% females, mean age 20.05). A confirmatory factor analysis confirmed the tri-dimensional structure of the English LMS in the Italian version.

**Results:**

The primary analysis found a significant negative correlation between mindfulness and psychological symptoms including obsessive-compulsive tendencies, depression, anxiety, and paranoid ideation. There was also a positive correlation between mindfulness and reports of quality of life.

**Conclusions:**

The Italian LMS appears reliable and it shows relevant correlations with well-being.

**Electronic supplementary material:**

The online version of this article (10.1186/s12955-018-0856-4) contains supplementary material, which is available to authorized users.

## Background

Langerian mindfulness is defined as the process of paying attention on purpose to the present moment, of being aware of novelty in experiences or situations, and of perceiving differences in contexts and events [1]. In contrast to the conceptualization of mindfulness associated with meditative practices that emphasizes a non-judgmental awareness of the present moment [2], Langerian mindfulness is characterized by a continuous creation of new categories, openness to new information and possibilities, awareness of more than one perspective, and flexibility in perspective-taking [3]. In Langer’s conceptualization, mindfulness is the opposite of mindlessness, the latter considering only a single perspective, of being entrenched in previous categorizations that do not incorporate new information from the current situational context. For the duration of this paper, when we use the term “mindfulness” we are referring to the Langerian framework. We use the term “meditative mindfulness” to refer to the framework developed by Kabat-Zinn.

The Langerian conceptualization of mindfulness is assessed through the Langer Mindfulness Scale (LMS). The LMS was originally developed with 21-items to assess four factors: novelty seeking, novelty producing, engagement, and flexibility [4]. These domains “describe a person’s relative openness to experience, willingness to challenge strict categories, and continual reassessment of the environment and their reactions to it” [4]. A 14-item version was later introduced with just three main factors: novelty seeking, novelty producing, and engagement. Pirson and colleagues [5] confirmed this tri-dimension structure in five independent studies with 4345 responses.

While clinical applications of Langerian mindfulness are underexplored, studies on meditative mindfulness demonstrate that state and trait mindfulness predict positive emotional states [6]. Additionally, individuals higher in meditative mindfulness tend to use functional stress management strategies [7]. Shapiro and colleagues [8] found that those participating in two meditation programs experienced mindfulness-mediated reductions in perceived stress and rumination compared to a waitlist control.

Similarly, the components of the Langerian mindfulness framework suggest clinical relevance, including psychological flexibility [9], attention to variability [10] and the ability to reframe negative experiences [11]. In our view, people with subclinical disorders or diagnosed with psychopathologies (including major depressive disorder) could benefit from a more mindful perspective of daily life. For example, drawing new distinctions between yesterday and today may help an individual combat feelings of hopelessness, a typical symptom of one subtype of depression called Hopeless Depression [12]. Specifically, a more mindful approach to change helps one realize that negative situations will not necessarily persist and that every moment presents an opportunity to notice new elements of a situation and take a different perspective about the current situation [1].

Researchers using the Five Facet Mindfulness Questionnaire have shown that meditative mindfulness (which they refer to as “dispositional mindfulness”) is positively correlated with positive self-appraisal [13]. Similarly, we expect that individuals high in Langerian mindfulness would be in the habit of reappraising negative situations and would be cautious of confining a complex situation to any one label/category.

Previous studies in our research group report a positive association between mindfulness as assessed by the LMS and quality of life (QOL) in people with Amyotrophic Lateral Sclerosis [14] and their caregivers [15]. Beyond these studies, little work has been done to understand the relationship between QOL and Langerian mindfulness. Therefore, the question remains: does the ability to flexibly interpret situations based on changing context and seek out novel experiences and alternative explanations correlate with well-being and reduced psychological symptoms? In order to provide an initial answer to that question, we designed a study that investigated the relationship between this measure of mindfulness and measures of well-being, including QOL and reduced psychological symptoms.

The study design included two aims. First we validated the tri-factor structure of the 14-item LMS in a sample of Italian students. Thus far, most work investigating Langerian mindfulness has been limited to the English version, though it has been translated into Malaysian [16] and Persian [17], with other translations pending, including Indian, Chinese, and Greek versions. Translating this scale into other languages should allow for future cross-cultural investigations of this construct. The validation should enable us to substantiate our primary objective, the exploration of potential relationships between Langerian mindfulness and psychological well-being.

## Methods

### Participants and procedures

The sample consisted of 248 second-year undergraduate students beginning a Clinical Psychology course at a private university in Milan, who were invited to voluntarily join the study by sending an email to one of the authors. The majority of the sample was composed of females (88.7%) and the average age was 21.05 years (S.D. = 2.84). Students were not familiar with the concept of mindfulness and had not received a structured mindfulness training. Students received an email containing a link to a survey composed of self-report questionnaires designed with the Qualtrics suite. Informed consent was obtained online prior to commencing the study.

The survey included demographic information and measurements of mindfulness, quality of life, and psychological symptoms. These variables were tested by the self-report questionnaires described below.

### Measures

#### Langer mindfulness scale

Mindfulness was assessed with the Italian version of the Langer Mindfulness Scale (LMS). The LMS is a 14-item questionnaire that assesses three domains associated with mindful thinking: novelty seeking, engagement and novelty producing. The LMS is a widely used instrument for the assessment of mindfulness with reliable psychometric validity [5]. The score ranges from to 14 to 98**,** with higher scores reflecting higher mindfulness. The Italian version is a translation from the original LMS. A back-translation process and an independent comparison with the English version were conducted to validate the consistency of the translation. The back-translation process did not lead to any changes in original item wording.

#### World Health Organization quality of life brief

The World Health Organization Quality of Life brief [WHOQoL-BREF; 18] was used to assess QOL. The WHOQOL-BREF is composed of 26 items. Two questions refer to quality of life and satisfaction with health, while the other 24 items are grouped into four domains: physical, psychological, social relationships, and environment. Participants rate their quality of life aspects on a 5-point scale over the last two weeks. Total score is the sum of all 26 items (maximum 130), with higher scores reflecting higher QOL. The scale has demonstrated good internal consistency (Cronbach’s alpha values for each of the four domain scores ranged from .66 to .84), and it was shown to be comparable to the WHOQOL-100 in discriminating between the ill and well groups [18]. We used the Italian version of the scale [19].

#### Symptom checklist 90-revised

Psychological symptoms were assessed with the Symptom Checklist 90-Revised [SCL-90-R; 20]. The inventory is composed of 90 items, each one of them representing a psychological symptom. Participants were required to assess the experience of each symptom in the past 2 weeks on a scale from 0 (Not at all) to 4 (Extremely). The checklist provides statistically reliable information about nine categories: Somatization, Obsessive Compulsive Disorder, Interpersonal Sensitivity, Depression, Anxiety, Hostility, Phobic Anxiety, Paranoid Ideation and Psychoticism. It also provides a Global Severity Index, designed to measure overall psychological distress. The scale has demonstrated good internal consistency, with Cronbach’s alpha values that range from .77 to .90 [20]. We use the validated Italian version [21].

### Statistical analysis

The factorial structure of the LMS was analyzed with a Confirmatory Factor Analysis (CFA), with a maximum-likelihood estimation method [22]. Internal consistency of the scale was assessed with Cronbach’s alpha. General LMS scores, as well as subscales, were correlated with the other outcome variables by Pearson’s *r*. The CFA was conducted using the package Lavaan for R (version 3.1.2), while the other statistical analyses were performed using SPSS software v.22. We compared a single-factor model with a three-factor model, based on the original LMS structure. We also tested a hierarchical model with a second order factor that was related to the three identified sub-scales. Goodness of fit was assessed with the following criteria: comparative fit index (CFI) > 0.9, standardized mean square residual (SMSR) closer value to 0, root mean square of approximation (RMSEA) value < .08 and the smallest AIC/BIC when comparing two or more models [22].

## Results

**Fig. 1:**
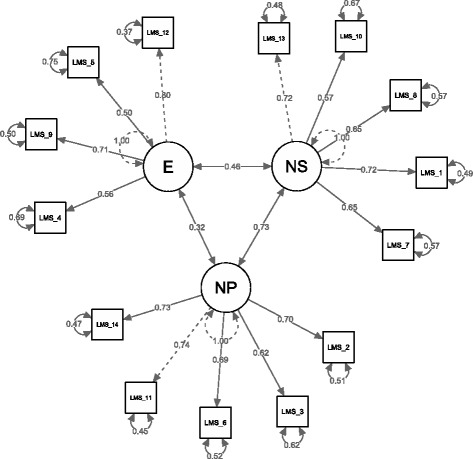
“Three factor model of the LMS. Coefficients are standardized loadings”. “Note: LMS = Langer Mindfulness Scale; E = Engagement; NS = Novelty Seeking; NP = Novelty Producing”

**Fig. 2:**
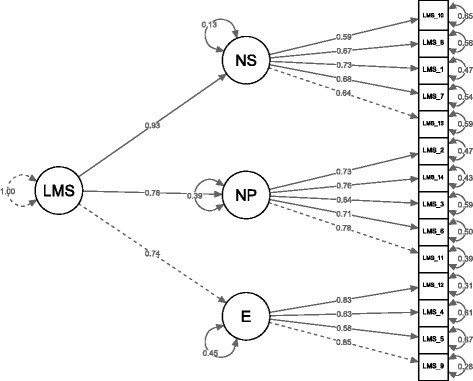
“Hierarchical model of the LMS - three factor structure. Coefficients are standardized loadings”. “Note: LMS = Langer Mindfulness Scale; E = Engagement; NS = Novelty Seeking; NP = Novelty Producing”

First, we tested a single-factor model in which all items contribute to an overall mindfulness factor. The fit of this model was: CFI = .20, TLI = .05, and RMSEA = .39, SRMR = .13., AIC = 10,619.83, BIC = 10,717.75. We then tested the three-factor model that was identified in the original version [4]. The three factors were allowed to inter-correlate. This model fit the sample well: CFI = .98, TLI = .97, and RMSEA = .07 (90% confidence interval: .05 to .08), SRMR = .08, AIC = 7816.49, BIC = 7924.91. The three-factor model proved a better fit index, including lower values of AIC and BIC. The factorial structure is reported in Fig. 1. Correlations among the LMS factors were all significant (ranging from .177 to .595, *p* < .01) and are reported in the supplementary materials (Additional file 1: Table S1). To test the validity of the total LMS score, we tested a second order factor model relating all the three components to a second order latent construct. We tested a hierarchical model with first order factors as the three sub-scales and a general second order factor (LMS total score, i.e., Mindfulness). The model fits the data properly, with CFI = .93, TLI = .80, and RMSEA = .09, SRMR = .27, AIC = 8889.14, BIC = 8903.54. The model is reported in Fig. 2.

The average value of the LMS composite score was 74.95 (*S.D*. = 10.17), while subscales means were as follows: Novelty Seeking, 27.65 (*S.D.* = 4.20); Novelty Producing, 23.59 (*S.D.* = 5.41); Engagement, 23.71 (*S.D*. = 3.72). The LMS scores did not differ significantly between male and female participants (*F*(1,242) = .53, *p* = .39) and were not associated with age (*r* = .01, *p* = .85). Cronbach’s coefficient *α* was .836, suggesting good internal consistency of the LMS. The internal consistency of the three sub-scales (i.e., novelty seeking, novelty producing, and engagement) was acceptable, with a Cronbach’s coefficient *α* of, respectively, .734, .821, and .777. The average item-total correlation was .471 (D.S. .098), indicating substantial within-scale common variance among items. The details of item-total correlations, mean, variance, and alpha-if-item deleted for each item are provided in the supplementary materials (Additional file 1: Table S2).

The LMS and its factors showed significant correlations with both the WHOQOL and the SCL-90. The overall LMS scores were positively correlated with WHOQOL overall QOL and general health, physical health, psychological health, and social relationships, while they were negatively correlated with the SCL-90 Global Severity Index and with the following SCL-90 factors: Obsessive-Compulsive, Interpersonal Sensitivity, Depression, Hostility, and Phobic Anxiety. Details and values of these correlations are reported in Tables 1 and 2.

**Table 1:** Correlations between LMS total scores and subscales and WHOQOL

		LMS Total	Novelty Seeking	Novelty Producing	Engagement
Overall Quality of Life and General Health	Pearson Correlation	.224^a^	.160^b^	.181^a^	.169^a^
	Sig. (2-tailed)	.001	.014	.005	.009
Physical Health	Pearson Correlation	.173^a^	.122	.153^b^	.113
	Sig. (2-tailed)	.007	.059	.017	.079
Psychological	Pearson Correlation	.326^a^	.300^a^	.236^a^	.208^a^
	Sig. (2-tailed)	.000	.000	.000	.001
Social relationships	Pearson Correlation	.151^b^	.093	.088	.178^a^
	Sig. (2-tailed)	.018	.147	.168	.005
Environment	Pearson Correlation	.029	.008	.012	.054
	Sig. (2-tailed)	.650	.901	.853	.408

*WHOQOL* World Health Organization Quality of Life brief, *LMS* Langer Mindfulness Scale

^a^ Correlation is significant at the 0.01 level (2-tailed)

^b^ Correlation is significant at the 0.05 level (2-tailed)

**Table 2:** Correlations between LMS total scores and subscales and SCL-90

		LMS Total	Novelty Seeking	Novelty Producing	Engagement
Global Score Index	Pearson Correlation	−.170^a^	−.069	−.084	−.270^a^
	Sig. (2-tailed)	.009	.290	.198	.000
Somatization	Pearson Correlation	−.066	.002	−.047	−.120
	Sig. (2-tailed)	.322	.973	.480	.073
Obsessive-Compulsive	Pearson Correlation	−.295^a^	−.204^a^	−.210^a^	−.269^a^
	Sig. (2-tailed)	.000	.002	.001	.000
Interpersonal Sensitivity	Pearson Correlation	−.195^a^	−.137^b^	−.129	−.192^a^
	Sig. (2-tailed)	.003	.038	.051	.003
Depression	Pearson Correlation	−.184^a^	−.140^b^	−.114	−.184^a^
	Sig. (2-tailed)	.005	.033	.084	.005
Anxiety	Pearson Correlation	−.089	.019	−.009	−.258^a^
	Sig. (2-tailed)	.180	.771	.897	.000
Hostility	Pearson Correlation	−.146^b^	−.067	−.060	−.242^a^
	Sig. (2-tailed)	.026	.310	.359	.000
Phobic Anxiety	Pearson Correlation	−.153^b^	−.004	−.041	−.360^a^
	Sig. (2-tailed)	.019	.949	.538	.000
Paranoid Ideation	Pearson Correlation	−.086	−.010	−.020	−.206^a^
	Sig. (2-tailed)	.194	.884	.768	.002
Psychoticism	Pearson Correlation	−.025	.090	.022	−.206^a^
	Sig. (2-tailed)	.711	.173	.737	.002

*LMS* Langer Mindfulness Scale, *SCL-90* Symptom Checklist 90-Revised

^a^ Correlation is significant at the 0.01 level (2-tailed)

^b^ Correlation is significant at the 0.05 level (2-tailed)

## Discussion

College students in Milan completed the Italian translation of the Langer Mindfulness Scale. Together with mindfulness, we assessed quality of life and psychological symptoms.

The original tri-dimensional factor structure of the LMS was confirmed. This suggests that the construct of mindfulness as developed by Langer [1] in the U.S. setting, could have similar components in the Italian context, supporting the intercultural validity of the construct. All three factors (Novelty Seeking, Novelty Producing, and Engagement) were correlated with each other, an indication that they all refer to the same mindfulness construct. That reflects the findings of the original version of the scale [4]. However, while the findings support a significant relationship between the 3 factors, these correlations are not perfect, suggesting that they also retain (or contribute) something distinctive and separate to the mindfulness construct.

As expected, Langerian mindfulness was positively associated with QOL and negatively associated with many psychological symptoms. Better physical and psychological well-being, as well as satisfying social relationships, tended to be associated with high mindfulness. Mindfulness resulted in a negative association with adverse psychological symptoms. In particular, it demonstrated a negative relationship with obsessive-compulsive symptoms, interpersonal sensitivity, depressive features, hostility, and phobic anxiety. Certain components of mindfulness provided higher negative correlations with psychological symptoms than others; specifically, the Engagement subscale was highly negatively associated with most psychological symptoms, and was also negatively associated with anxiety, paranoid ideation, and psychoticism. This finding is not particularly surprising, since people with higher levels of adverse psychological symptoms (e.g., depression, anxiety, and also dissociative features) tend not to engage in life experiences or activities. In this way, psychological problems can be interpreted in terms of mindlessness, which is the opposite of mindfulness, remaining entrenched in previous established categories is one of the main characteristics of anxiety and depression, and of obsessive thoughts. These categories are what the cognitive-behavioral therapy approach refers to as irrational thoughts [23]. On the other hand, a mindful attitude can promote psychological adaptation, with an openness to new information, resulting in higher flexibility and resilience [1]. It has been previously indicated that these attitudes reduce psychological distress and improve QOL and psychological well-being [9]. A mindful attitude could therefore promote well-being by reducing distress and helping solve psychological problems. The impact of this mindfulness construct appears to result in greater psychological well-being, better physical health, and improved social relationships. In this sense the entire bio-psycho-social model of the person [24] can be influenced by mindfulness.

The interpretation of the direction of the results was theory-driven, in line with our hypothesis. However, given the correlational design, it could also be argued that psychological symptoms and low QoL promote mindlessness, or that all these constructs depend on another third variable. The design and the inferences that can be drawn from it constitute a study limitation. Strong inferences about the causal direction should be drawn from studies with an experimental design. Randomized controlled trials about Langerian mindfulness, QOL and psychological well-being are warranted to explore what seems to be a promising association. Another limitation concerns the instrument’s external validity. Our data reflected undergraduate students in psychology, with a large presence of female participants. They joined the study before the concept of mindfulness was explained and were blind to the project’s hypothesis. However, we cannot assume that the conclusions can be extended to the entire population (despite the common procedure of including students in scientific studies and extending the results to the general population). Another danger to the external validity is the level of distress that is reported by the sample of students. This is in line with previous studies that found students to be more distressed than the general population [25]. These issues suggest the need for further studies to verify the extension of these results to different populations.

## Conclusions

Despite a few limitations, this is the first study to our knowledge that explores the connection between Langerian Mindfulness, psychological symptoms and quality of life, with the potential exception of people with Amyotrophic Lateral Sclerosis [14]. Our results suggest by increasing mindfulness as reflected in the Langerian construct, both QOL and psychological well-being will improve. The theoretical construct of mindfulness according to Langer does not require meditation or similar forms of training to be increased [26]. That is in line with the findings from other studies that showed changes on some mindfulness scales conducting informal practices such as dishwashing [27]. One becomes more mindful by maintaining openness and attention to novelty throughout one’s daily life. Mindfulness is a skill that can be improved by small cognitive exercises that do not require an extensive time investment [28, 29]. The simplicity of the approach, easily applicable in different contexts, could helpfully inform future clinical and social applications.

## Additional file

Additional file 1: Table S1. Correlations among LMS factors and LMS total score. **Table S1.** Item-total statistics. (DOCX 55 kb)

## Abbreviations

AIC: Akaike information criterion
BIC: Bayesian information criterion
CFA: Confirmatory Factor Analysis
CFI: comparative fit index
LMS: Langer Mindfulness Scale
QOL: Quality of Life
RMSEA: root mean square of approximation
SCL-90-R: Symptom Checklist 90- Revised
SMSR: standardized mean square residual
TLI: Tucker-Lewis index
WHOQOL-BREF: World Health Organization Quality of Life brief
