# Supplementary material for: Langerian mindfulness, quality of life and psychological symptoms in a sample of Italian students
Source: Health Qual Life Outcomes. 2018 Feb 6;16:29. doi: 10.1186/s12955-018-0856-4 (PMC5801901; doi:10.1186/s12955-018-0856-4)
Supplement: Additional file 1: Table S1. — Correlations among LMS factors and LMS total score. Table S1. Item-total statistics. (DOCX 55 kb) [file 12955_2018_856_MOESM1_ESM.docx]

**Table S1. Correlations among LMS factors and LMS total score**

|  |  | LMS Total | Novelty Seeking | Novelty Producing | Engagement |
| --- | --- | --- | --- | --- | --- |
| LMS Total | Pearson Correlation | 1 |  |  |  |
|  | Sig. (2-tailed) |  |  |  |  |
| Novelty Seeking | Pearson Correlation | .829** | 1 |  |  |
|  | Sig. (2-tailed) | <.001 |  |  |  |
| Novelty Producing | Pearson Correlation | .842** | .595** | 1 |  |
|  | Sig. (2-tailed) | <.001 | <.001 |  |  |
| Engagement | Pearson Correlation | .573** | .273** | .177** | 1 |
|  | Sig. (2-tailed) | <.001 | <.001 | .006 |  |

Note: ** Correlation is significant at the 0.01 level (2-tailed). LMS = Langer Mindfulness Scale

**Table S2 – Item-total statistics**

|  | Corrected Item-Total Correlation | Scale Mean if Item Deleted | Scale Variance if Item Deleted | Cronbach's Alpha if Item Deleted |
| --- | --- | --- | --- | --- |
| LMS 1 | .526 | 69.213 | 91.131 | .824 |
| LMS 2 | .596 | 69.946 | 85.071 | .817 |
| LMS 3 | .481 | 70.422 | 89.792 | .826 |
| LMS 4 | .332 | 68.995 | 94.342 | .835 |
| LMS 5 | .378 | 68.877 | 93.458 | .832 |
| LMS 6 | .514 | 70.360 | 87.038 | .823 |
| LMS 7 | .469 | 69.020 | 93.609 | .827 |
| LMS 8 | .554 | 69.791 | 89.705 | .821 |
| LMS 9 | .324 | 69.250 | 94.098 | .835 |
| LMS 10 | .514 | 69.922 | 86.752 | .824 |
| LMS 11 | .588 | 70.658 | 86.637 | .818 |
| LMS 12 | .332 | 68.995 | 94.342 | .835 |
| LMS 13 | .416 | 69.192 | 92.329 | .830 |
| LMS 14 | .571 | 69.815 | 86.036 | .819 |

Note: LMS = Langer Mindfulness Scale
